# Depositional environment of the late Paleocene-early Eocene Sinjar Formation, Iraq: Implications from facies analysis, mineralogical and geochemical proxies

**DOI:** 10.1016/j.heliyon.2024.e25657

**Published:** 2024-02-10

**Authors:** Noor T. Al-Taee, Ali I. Al-Juboury, Imad M. Ghafor, Giovanni Zanoni, Harry Rowe

**Affiliations:** a Geology Department, College of Science, Mosul University, Iraq; b Al-Kitab University, Kirkuk, 36015, Iraq; c Geology Department, Sulaimaniya University, Iraq; d RohmTek, Houston, TX, 77024, USA; e Premier Corex Laboratories, Houston, TX, 77041, USA

**Keywords:** Facies analysis, Sinjar Formation, P–E boundary, Paleoredox, Paleoproductivity, Paleoclimate, Paleosalinity, Depositional environment, Iraq

## Abstract

Reconstruction of the depositional environment of the Paleocene-Eocene Sinjar Formation from two outcrop sections in northwestern and northeastern Iraq has been evaluated using the traditional petrographic and facies analysis supported by X-ray diffraction and scanning electron microscopy with a focus on the Paleocene-Eocene (P-E) transition boundary. To this end, major and trace elemental geochemistry was conducted and various paleoenvironmental proxies for the paleoredox, paleoclimate, paleosalinity and paleoproductivity were determined in order to evaluate the changes in widely acknowledged environmental and climatic indicators and the elemental enrichment/depletion across the P–E boundary. The redox-sensitive trace element enrichment and the ir ratios (V/V + Ni, V/Cr, and U/Th) indicate that normal oxygenated circumstances prevailed during the late Paleocene deposition, and that anoxic conditions and a gradual commencement of oxygen depletion occurred during the early Eocene deposition. The coeval increase in the P_2_O_5_ content, P/Ti, and P/Al ratios in the Eocene sediments suggests an increase in nutrients and primary productivity due to the effect of upwelling currents during early Eocene. The conditions can be verified by observing a small change in salinity levels from low to high across the P-E boundary, which can be indicated by the Sr/Ba ratios. In addition, certain minerals such as Mg-calcite, dolomite, and palygorskite are commonly present, and paleoclimatic changes can be observed across the P-E transition from arid to semiarid and then to humid conditions, which can be recorded from C-values, Sr-Cu, Rb/Sr ratios, and clay mineralogy. These conditions were noted in the Sinjar Formation, which is made up of many microfacies such as lime mudstone, wackstone, packstone, grainstone and boundstone. These microfacies were deposited in a shallow marine environment that extended from tidal flats to reef slopes, with a developed reef environment that included back reef, reef core, and fore reef environments.

## 1 Introduction

The Paleocene-Eocene (P-E) period includes several events that are regionally recognized, such as the Paleocene-Eocene Thermal Maximum (PETM) and the Oceanic Anoxic Events (OAEs) that are characterized by various climatic and paleoenvironmental changes affecting the sedimentary environments and their bio-content due to change in worldwide carbon and biochemical cycles [1].

The Paleocene-Eocene boundary is characterized by various biological, mineralogical, and geochemical factors that explain the global paleoenvironmental changes, including significant climatic changes. These changes include a strong negative carbon isotope excursion (ranging from −2 to −7‰) as reported by Ref. [2], an increase in water salinity, a decrease in oxygen levels, a shoaling of the calcite compensation depth (CCD), the extinction of approximately 50% of benthic foraminifera, and a turnover in most of the calcareous nannofossils and planktic foraminifera [[3], [4], [5], [6], [7], [8], [9], [10]].

In the current study, two outcrop sections of the late Paleocene-early Eocene Sinjar Formations at Kalka Smaq, near Dokan area of northeastern Iraq and the type section of Sinjar Formation at Sinjar area of northwestern Iraq, have been chosen and subjected to a detailed microfacies analysis using traditional microscopic analysis supported by scanning electron microscopic (SEM), X-ray diffraction (XRD), and elemental X-ray fluorescence (XRF) analyses.

The Sinjar Formation is predominantly made up of well-layered limestone, which has undergone recrystallization and represents the shallow marine reefal and lagoonal deposition. The studied Sinjar Formation has been described in numerous papers as well as several books and theses (e.g., Refs. [[11], [12], [13], [14], [15], [16], [17], [18], [19]]. Most of these studies were focused on biostratigraphy and lithostratigraphy of the Sinjar Formation without a detailed study of the depositional environment using a multidisciplinary sedimentological, mineralogical and geochemical proxies and the relation to Paleocene-early Eocene events from northern Iraq.

The present study aimed to characterize the depositional environment of the Sinjar Formation through litho- and microfacies analysis, mineralogical examination, and geochemical data. The investigation focused on identifying the paleoenvironmental changes that occurred across the Paleocene-Eocene (P-E) boundary in northeastern and northwestern Iraq.

## 2 Geological setting

The Paleocene-early Eocene formations in northern Iraq are represented by the Kolosh, Khurmala, Sinjar, Avanah, Gercus and Pila Spi formations that are distributed in various localities in northern, northeastern and northwestern Iraq in either outcrops or have been penetrated by several wells in the oilfield located in these parts of Iraq. The Paleocene to early Eocene period represents the middle part of the Arabian Plate Tectonostratigraphic Megasequence AP10 [20].

Towards the end of the Paleocene, the Tethyan Ocean was an extensive epicontinental basin that was deepening northward, encompassing most of the Arabian Craton including Iraq and neighboring areas. The southern margin of the Tethys was strongly affected by intermittent upwelling episodes due to its location in the northern tropical zone [21,22]. In the region, multiple stratigraphic sections exhibit biotic and geochemical evidence associated with upwelling episodes, showing comparable and unique characteristics that indicate significant global changes during the Paleocene-Eocene period [23].

**Fig. 1:**
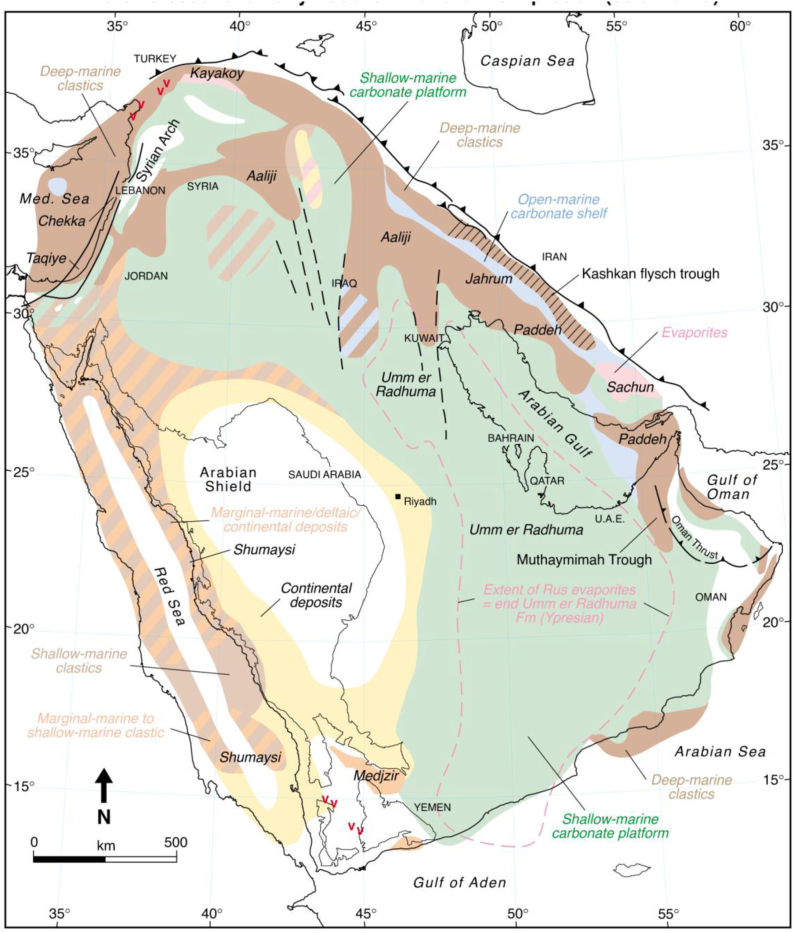
Late Paleocene to Early Eocene (Thanetian to Ypresian 60.9–49 Ma) paleofacies map, after [24], illustrating deposition of shallow-marine platform carbonates (Sinjar Formation) interfingering with deep-marine clastics and carbonates of the Aaliji Formation.

The studied formation is regarded as one of the most important formations in the Paleocene-lower Eocene cycle. During this period, Shallow-marine carbonates onlapped far onto the Arabian Craton with a maximum flooding [Pg10] being reached in the Early Eocene [24]. Deposition was characterized by calcarenitic limestones and dolomites dominated by a variety of pelecypods and gastropods that represent deposition in a protected to lagoonal depositional environment (Fig. 1).

The Cenozoic sedimentary cycle, which spans across southern and northeastern Turkey, northern Iraq, and northwestern Iran, is mainly comprised of a cyclic succession of sediments. The Sinjar Formation, a reefal limestone unit, represents the shallowest portion of this cycle and can reach up to 176 m in thickness in the Sinjar anticline. However, towards the eastern end of the anticline, its thickness gradually decreases and it laterally interfingers as a tongue with the deeper facies of the Aaliji Formation.

The Paleocene-lower Eocene cycle is the first sedimentary cycle of the Cenozoic after the regional transgression covered Iraq and neighboring countries at the beginning of Cenozoic due to Alpine orogeny. Two ridges characterized this sedimentary cycle, the first extended from Amadiya in the northernmost part of Iraq through Ranya, to Halabja in northeastern Iraq (including the studied Kalka Smaq section) where the flysch sediments of the Kolosh Formation were also deposited [25]. While the second ridge is located in northwestern Iraq, where the shallow marine Khurmala and shallow reefal Sinjar sediments were deposited. These sediments were laterally changed at the end of the cycle to shallow sediments rich in benthonic foraminifera that interfinger in the northeastern part of Iraq with the Kolosh flysch sediments while in northwestern and central parts of Iraq, they were interfingering with deep marine marl sediments of the Aaliji Formation [14]. A regional regression covered the area at the end of the Paleocene-lower Eocene cycle and was indicated by common stromatolite structures at the top of the Sinjar Formation [14,15].

**Fig. 2:**
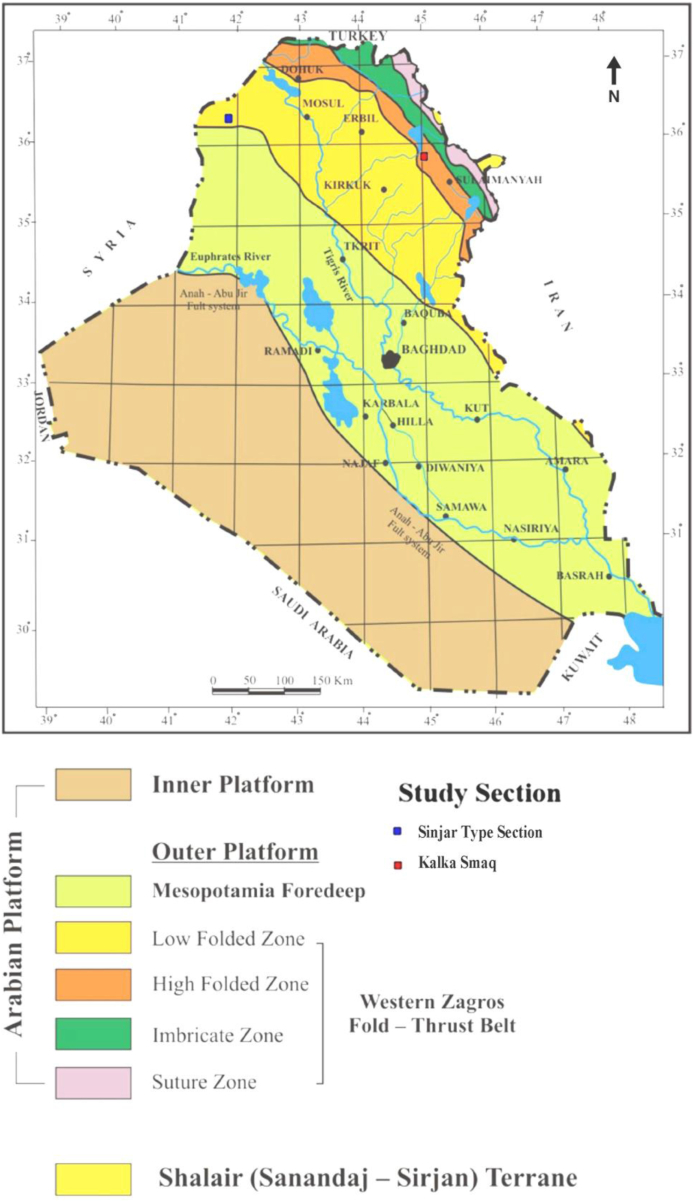
Tectonic divisions of Iraq, after [26] and the location of the studied sections.

Tectonically, both sections lie in the northeastern part of the Arabian Plate. According to the tectonic map of Iraq [26], Sinjar section lies in the Low Folded Zone, while the Kalka Smaq section is located in the High Folded Zone on the Unstable shelf of Arabian Plate [27], (Fig. 2).

## 3 Materials and methods

The present work is based on field and laboratory examination of the late Paleocene-early Eocene Sinjar Formation in northeastern and northwestern Iraq. A total of 241 samples (125 samples from Kalka Smaq section and 116 from the type Sinjar section) were selected from the two studied sections for the petrographic, mineralogical and geochemical studies. Thin sections were made from (179 samples, 79 samples from Kalka Smaq section and 100 samples from the type Sinjar section) and then examined to assign microfacies types, diagenetic processes, and depositional models. The carbonate facies and textures were distinguished following classification of Dunham [28] and its modification by Ref. [29]. Comparison with Standard Microfacies (SMF) types, and facies zones of [30,31] respectively were conducted. Scanning electron microscopy (SEM) analyses were completed on selected samples at Premier Corex Laboratories in Houston, U.S.A., employing an FEI Quanta FEG 650 FE-SEM device fitted with an FEI R580 Everhart-Thornley (ETD) electron detector and two Bruker EDS XFlash 5030 energy dispersive X-ray spectroscopy (EDS) detectors. For the investigation, freshly broken surfaces were produced by breaking a fragment of rock as close to perpendicular to the bedding as possible. Using conductive high-viscosity glue, a few samples were mounted on aluminum stubs from the Kalka Smaq section. Before being inserted into the SEM, they were sputter coated with 10 nm of iridium using a Leica EM ACE600 sputter coater. The measurements used an accelerating voltage of 10 kV and were carried out in a high vacuum.

X-Ray diffraction (XRD) mineralogy analysis was performed on selected (11) samples from the Kalka Smaq section at Premier Corex Laboratories in Houston, U.S.A. on bulk rock samples using a Bruker D8 Advance XRD instrument equipped with a theta-theta goniometer with a 250 cm radius and a Lynxeye XE-T detector. All measurements were performed using CuK radiation, voltage and current were 40 kV and 30 mA, respectively on <300 μm powdered whole rock samples. The powdered material was further milled in a McCrone XRD mill for 13 min, which ensures a narrow grain size distribution, and brings the average grain size down to 10–20 μm. Quantification of mineral phases in the bulk diffraction pattern is accomplished using the TOPAS software package. The results of XRD analysis are summarized in Table (1).

The XRF analysis was done on (24) samples using a Bruker Tracer 5i XRF instrument installed at Premier Corex Laboratories in Houston, U.S.A. The instrument utilizes a Rh tube and three separate phases of analysis are done in order to measure the full suite of elements. Firstly, measurement is done at the energy settings of 10 kV 35 μA for a duration of 10 s under a helium atmosphere. This phase produces a spectrum that is well suited for measurement of the elements with lower energies (Na, Mg, Al, Si, P, S, K, Ca, Ba, Ti, V, Cr, Mn, Fe). The second phase of analysis is at a higher energy level (45 kV 40 μA), has a longer count time (30 s) and utilizes a filter with a thickness of 25 μm of Ti and 300 μm Al. This phase of analysis is designed to enhance the signal from the following trace elements: Co, Ni, Cu, Zn, Ga, Pb, As, Rb, Th, Sr, Y, Zr, Nb, and Mo. Lastly, a third phase of analysis is done in order to attain a better analysis on some of the trace elements, mainly U. The third phase has an energy level of 44 kV and 60 μA with a 90 s count time and a filter with 100 μm Cu, 25 μm Ti and 300 μm Al. Table (2) illustrates the accuracy and the lower detection limit for the major and trace elements used in the currents study.

## 4 Results and discussion

### 4.1 Lithostratigraphy

**Fig. 3:**
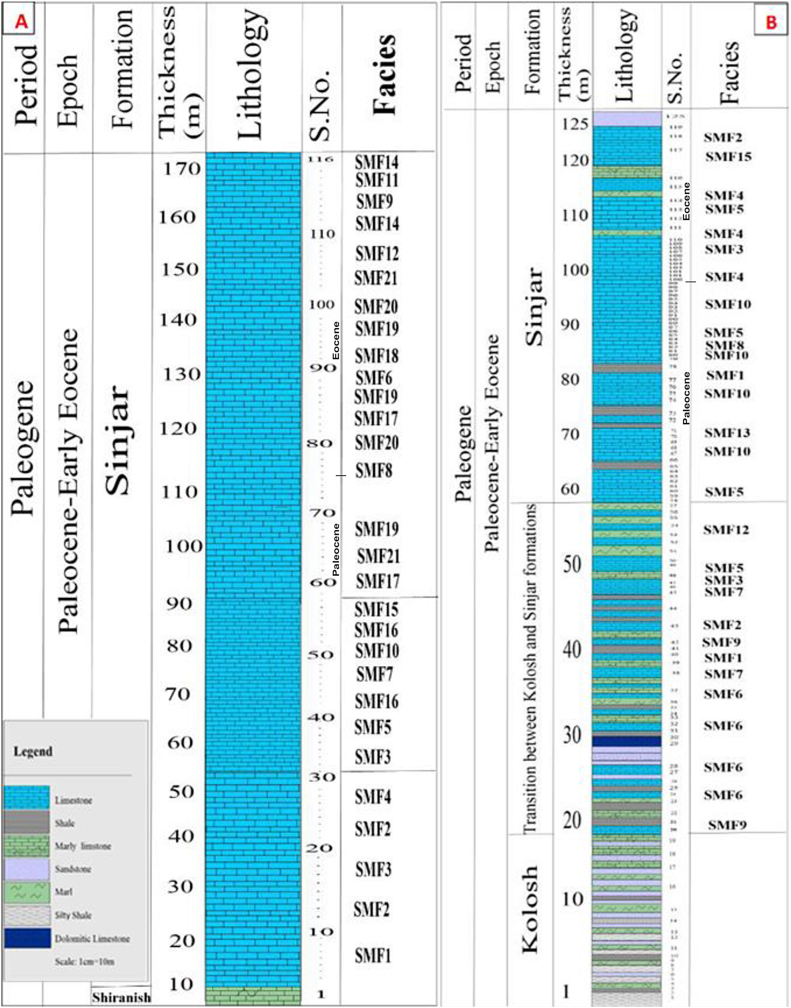
Lithological log showing microfacies succession of the Sinjar Formation exposed at the type section at Sinjar area (A) and Kalka Smaq area (B).

**Fig. 4:**
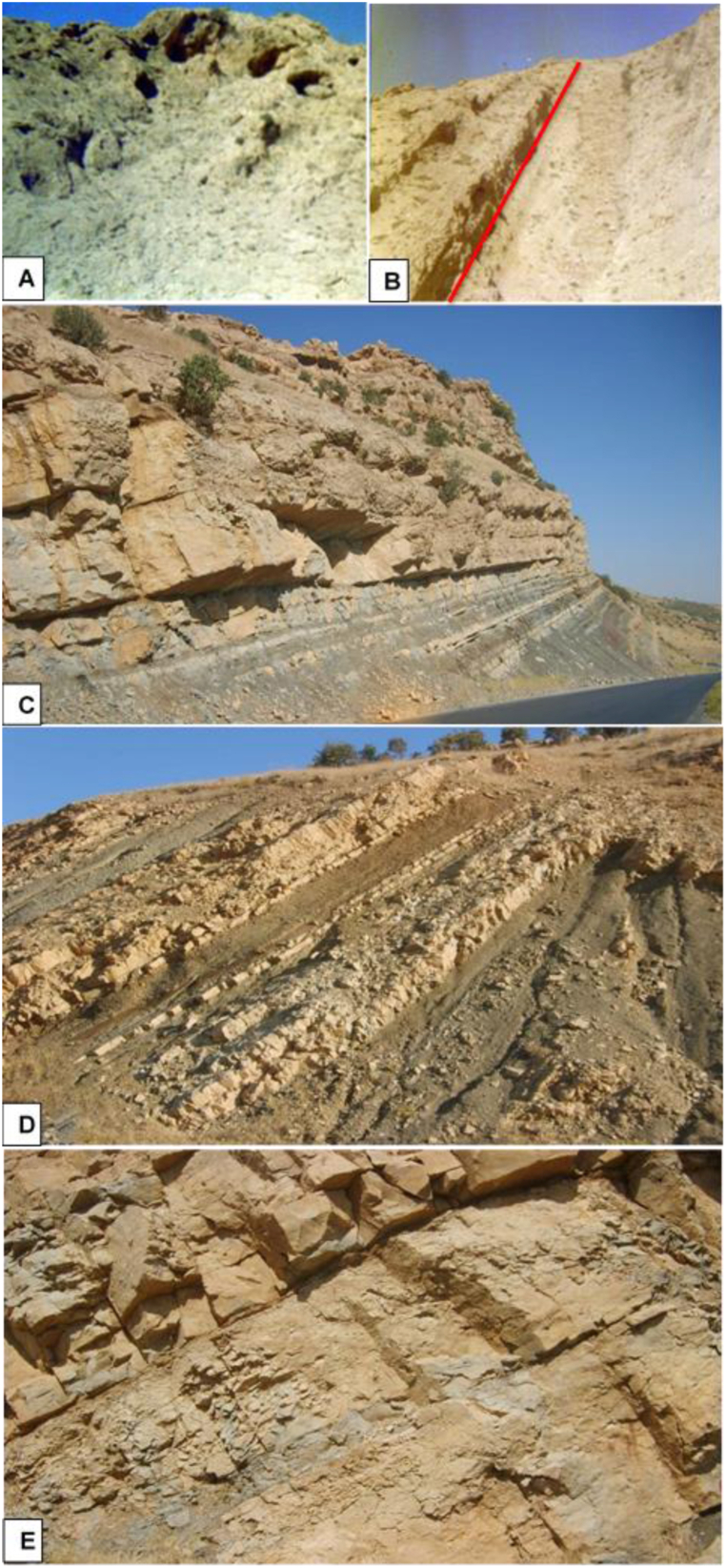
Field photograph of Sinjar Succession at type section showing the lower massive bedded unit (A) and the middle unit of well bedded limestone separated from the upper unit that forms high ridge of hard massive bedded limestone (B). C- represent the well bedded marly limestone and marl with overlying massive bedded limestone at Kalka Smaq section. D- overview of intercalation of marl, marly limestone and shale in the middle part of Sinjar Formation at Kalka Smaq section and E- massive bedded, fractured and cavernous limestone in the uppermost part of Sinjar Formation at Kalka Smaq section.

The Sinjar Formation, located at the Sinjar anticline in northwestern Iraq, consists of a limestone succession approximately 170 m thick. Based on the type of bedding, the formation can be divided into three stratigraphic units (Fig. 3A). The lower unit, spanning about 45 m, is characterized by sporadically medium bedded, massive, recrystallized, yellowish-white limestone with a thickness of 4–6 m. The unit also features common gastropod shells, and is cavernous in nature (Fig. 4A). The middle unit, which comprises approximately 38 m in the type locality of the formation, is composed of well-bedded limestone with 40–80 cm of the beds. This unit is characterized by weathering due to its lower hardness compared to the other units and contains partially recrystallized limestone. Large benthonic foraminifera shells, bivalves, and bioturbation are commonly observed in this unit (Fig. 4B). The upper unit, with a thickness of about 87 m, is characterized by massive and bedded limestone, forming a high ridge due to its hardness (Fig. 4B). These limestone beds are yellowish-white to gray in color and are rich in benthonic foraminifera (such as nummulites) and large gastropod shells. Additionally, dome-structures of red algae can also be observed in this unit.

**Fig. 5:**
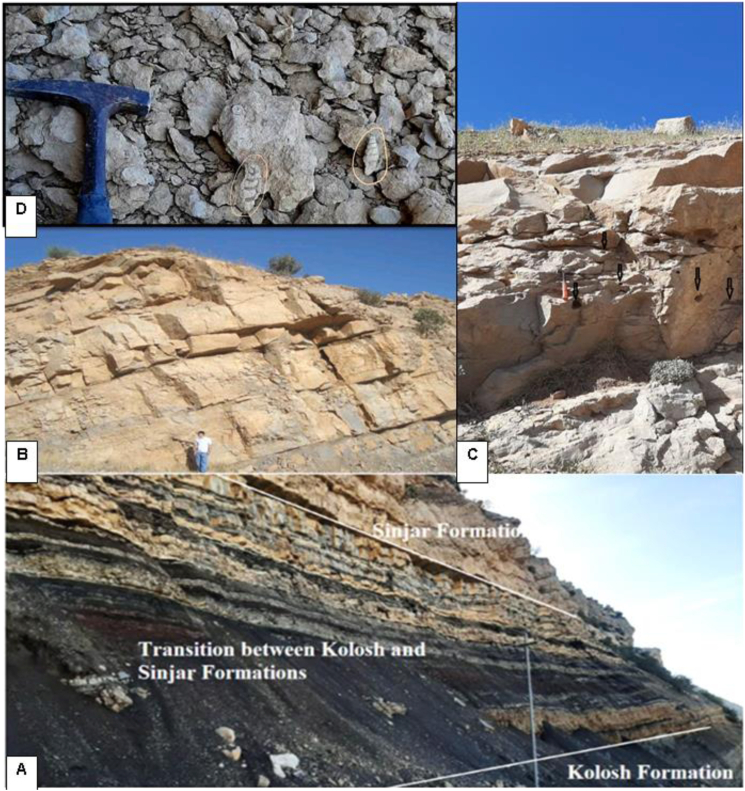
Field photograph of the lower transitional zone between the Kolosh and Sinjar formation at Kalka Smaq section (A) represented by dark shale, silty shale, sandstone and marl. B- High ridge of hard, massive recrystallized fractured limestone of Sinjar Formation at Kalka Smaq section. C- Cavernous limestone of Sinjar Formation at Kalka Smaq section. D- Gastropod shells in marl of Sinjar Formation at Klka - Smaq section.

On the other hand, Sinjar Formation in the Kalka Smaq section at Sara Anticline, near Dokan area of northeastern Iraq (Fig. 3B), is composed of 105 m as tongues of gray soft shale with a thickness of one to 2 m interfingering with marl, marly limestone, and white limestone beds in the transition between the Kolosh and Sinjar formations. The total thickness of the studied transition zone reaches up to 37 m (Fig. 4, Fig. 5A). The transition zone overlaid by limestone of the Sinjar Formation, with a total thickness of 7 m, yellowish white in color (Fig. 4C), followed by 26 m of interbedded shale, marl, well bedded marly limestone and dolomitic limestone (Fig. 4D). The upper part of the Kalka Smaq section including 35 m of a massive bedded fractured limestone and marly limestone (Fig. 4, Fig. 5B) rich in cavities, bioturbidites and gastropod shells (Fig. 5C and D).

### 4.2 Petrographic components, mineralogy and diagenesis (Fig. 6, Fig. 7, Fig. 8, Fig. 9, Fig. 10, Fig. 11, Fig. 12)

Petrographic study is based on 179 samples of limestone, marly limestone and few units of dolomitic limestone from the Sinjar Formation in both studied sections at the type section and Kalka Smaq section.

The dominated components are represented by skeletal carbonate particles that are composed of benthic foraminifera, miliolids, *Nummulites*, *Disocyclina* - and *Textularia* -along with echinoids, green algae, gastropods, cephalopods, pelecypods, bryozoans, and coral algae. Moreover, the studied rocks contain non-carbonate components such as quartz, chert, pyrite, and iron oxides. Various diagenetic events have affected these rocks, including recrystallization, compaction, cementation, micritization, dolomitization, neomorphism, silicification, bioturbation, dissolution, pyrite authigenesis, fenestral and moldic porosity, and stylolite formation. XRD analysis (Table 1) has revealed that calcite and Mg-calcite are the main carbonate constituents in addition to dolomite and Fe-dolomite. Quartz, traces of plagioclase and clay minerals represented by smectite, illite-mica, palygorskite and traces of chlorite beside trace traces of talc in the transition zone and serpentine are all minor phases. Sporadic pyrite and hematite were also recorded in few samples.

**Table 1:** XRD data for the marl limestone and limestone from the transition zone between Kolosh and Sinjar formation (SK sample and Sinjar Formation (S samples), Kalka Smaq section. Clay minerals, S = Smectite, I = Illite-mica, C = Chlorite, P = Palygorskite, T = Talc, Sr=Serpentine, Tr = Trace amount. (See Fig. 3B for samples location).

Sample number	Framework silicates		Clay	Carbonates				Others		Total	Clay minerals						
	Quartz	Plagioclase		Calcite	Mg-calcite	Dolomite	Fe-dolomite	Pyrite	Hematite		S	I	C	P	T	Sr	Total
S119	5.6	0.4	2.5	1.7	4.2	74.6	11.0	0.0	0.0	100.0	1.6	0.3	0.2	0.0	0.0	0.4	2.5
S113	5.4	0.0	1.9	62.1	30.2	0.1	0.3	0.0	0.0	100.0	0.5	0.8	0.6	0.0	0.0	0.0	1.9
S110	0.9	0.0	0.6	20.4	69.6	2.5	6.0	0.0	0.0	100.0	0.6	0.0	0.0	0.0	0.0	0.0	0.6
S108	1.8	0.0	0.8	19.4	77.5	0.2	0.3	0.0	0.0	100.0	0.8	0.0	0.0	0.0	0.0	0.0	0.8
S100	2.9	0.0	0.6	13.8	82.3	0.1	0.3	0.0	0.0	100.0	0.6	0.0	0.0	0.0	0.0	Tr	0.6
S90	1.0	0.0	0.6	26.8	70.6	0.4	0.6	0.0	0.0	100.0	0.6	0.0	0.0	0.0	0.0	0.0	0.6
S80	1.0	0.0	0.6	15.5	81.0	0.5	1.4	0.0	0.0	100.0	0.6	0.0	0.0	Tr	0.0	Tr	0.6
S71	1.3	0.0	3.7	13.4	80.0	0.4	1.1	0.1	0.0	100.0	0.8	0.0	0.0	2.7	0.0	0.2	3.7
S66	1.4	0.0	0.8	9.4	83.2	2.0	3.0	0.2	0.0	100.0	01	0.5	0.0	0.0	0.0	0.2	0.8
S58	4.2	0.0	3.5	18.0	40.0	20.0	12.5	0.8	1.0	100.0	1.6	0.0	0.4	1.5	0.0	Tr	3.5
SK52	12.5	0.1	4.6	0.5	0.9	35.9	44.8	0.7	0.0	100.0	2.0	0.0	0.8	0.0	1.8	0.0	4.6

SEM study shows that calcite is present in several forms; grainy calcite cement and syntaxial cement around echinoids or euhedral (rhombohedral), microcrystals, star-shaped, and columnar crystals. Dolomite presents in the well crystalized rhombic shapes accompanying mostly with columnar calcite and fibrous palygorskite.

The studied samples were taken across Paleocene–Eocene (P-E) boundary within Sinjar Formation at Kalka Smaq section (Fig. 3B, Table 1). Samples 58–99 from the Paleocene (Thanetian) while samples 100–119 from the Eocene (Ypersian), [32].

The XRD results (Table 1) show an increase in the amount of quartz in Eocene interval samples accompanying with increase in clay mineral content (with a considerable increase of illite) and decrease in the amount of Mg-calcite which is recorded as the main carbonate minerals in the XRD diffractograms (see Fig. 7, Fig. 11C).

### 4.3 Facies analysis

Based on various sedimentological and biological features, the Sinjar Formation is divided into five microfacies.

#### 4.3.1 Lime mudstone microfacies

This facies is present in the lower massive unit at the type section in Sinjar and in the lower transitional zone in Kalka Smaq section (Fig. 3B). It consists of a micritic groundmass with less than 10% grains. Based on the type of grains, it is further divided into two submicrofacies.

##### 4.3.1.1 Unfossiliferous lime mudstone submicrofacies (SMF1)

**Fig. 6:**
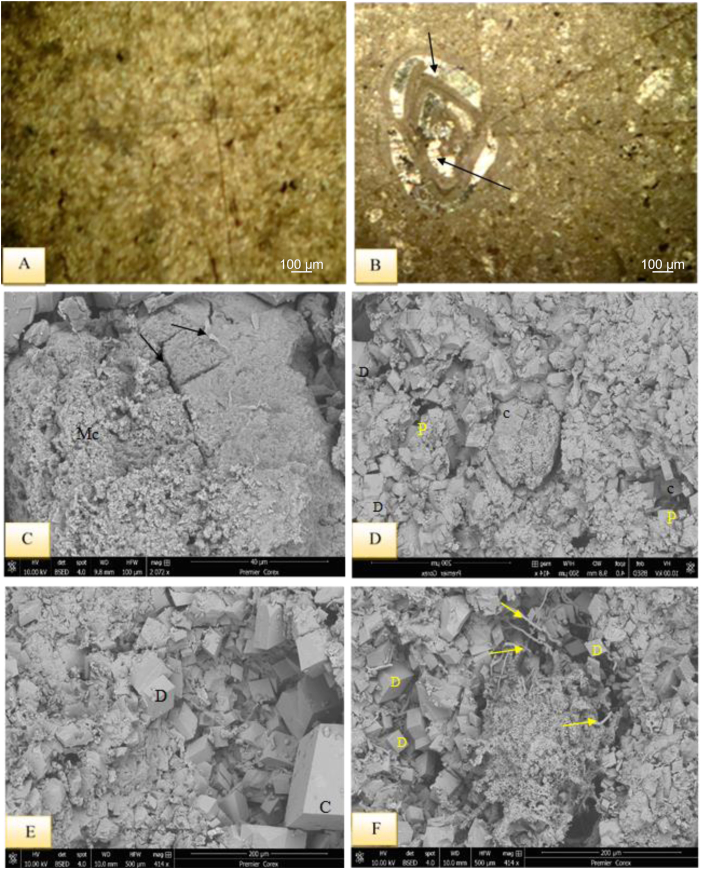
A- Unfossiliferous Lime Mudstone Submicrofacies with micritic groundmass, sample 77, Sinjar Formation, Kalka Smaq section. B- fossiliferous Lime Mudstone Submicrofacies with miliolid and scattered pyrite grains, note cementation within miliolid shell (arrows), sample 79, Sinjar Formation, Kalka Smaq section. SEM images from sample 46, Sinjar Formation, Kalka Smaq section showing C- fine micritic grains (Mc) and stylolite (arrows), D- common calcite (C), dolomite (D), and pores (P) due to dissolution process, E- enlarged view of D illustrates cementation by hexagonal calcite (C) and rhombic dolomite crystals (D), F- Palygorskite fibers (arrows) accompanied with dolomite (D). See Fig. 3 for samples location.

This microfacies is characterized by micritic groundmass with very rare skeletal grains and their clasts (Fig. 6A). The main diagenetic events include, recrystallization, stylolite formation, cementation, dolomitization in the form of aphanotopic texture and porosity. The studied limestones are well bedded with no bioturbation. This microfacies is recorded from the Sinjar Formation in both the Kalka Smaq and type section.

##### 4.3.1.2 Fossiliferous lime mudstone submicrofacies (SMF2*)*

Miliolids and *Textularia* - are the benthonic foraminifera in micritic groundmass along with echinoids, green algae, bivalves and some external clasts such as chert, quartz and iron oxides form the main constituents of this microfacies (Fig. 6B).

##### 4.3.1.3 Interpretation

The presence of un-fossiliferous carbonate mud with no bioturbation is the common feature of restricted platform interiors as a barren lagoon on the back reef that is unconnected to the sea water and high salinity [[33], [34], [35]]. This is indicated by common calcite and dolomite accompanied with by palygorskite fibers (Fig. 6C–F). Warm, saline evaporitic conditions with alkaline fluids rich in Si, Al, and Mg are suitable for authigenic palygorskite formation accompanied with dolomite [36,37]. The presence of small-sized benthic foraminifera along with bivalves, echinoids and green algae, and some external clasts may also refer to shallow marine and restricted setting with high salinity. Both facies fall under the FZ-8 SMF, 2–3 of the [30,31] standard microfacies type respectively, and suggest deposition on restricted platform interior (back reef settings).

#### 4.3.2 Lime wackestone microfacies

This microfacies typically exhibits 10–50% of skeletal grains and is predominantly composed of mud. Its color ranges from pale brown to dark brown, particularly when clay minerals and organic matter are present. It can be further subdivided into six submicrofacies.

##### 4.3.2.1 Bioclastic lime wackestone submicrofacies (SMF3)

**Fig. 7:**
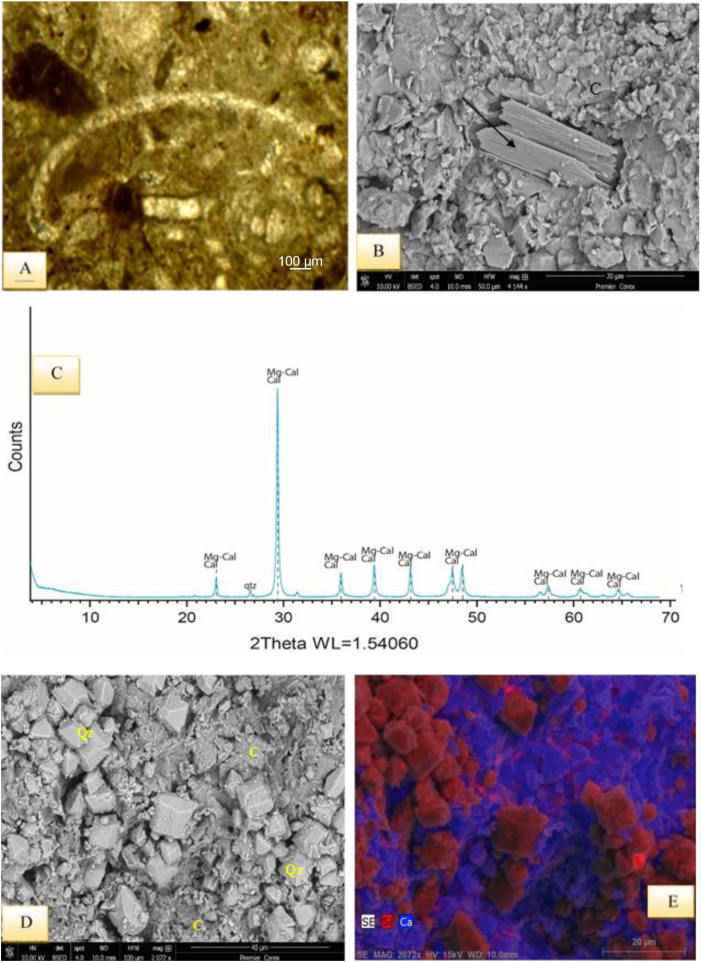
A- Bioclastic Lime Wackestone Submicrofacies, sample 82, Sinjar Formation, Kalka Smaq section. B- SEM image of bioclastic lime wackestone showing algae fragment (arrow) in micritic and sparry groundmass formed of calcite (C), sample 108, Sinjar Formation, Kalka Smaq section. C- XRD diffractogram showing calcite and Mg-calcite as dominating carbonate minerals, sample 108, Sinjar Formation, Kalka Smaq section. D- SEM image of quartz authigenesis in the form of dodecahedron quartz (QZ) and presence of calcite (C), sample 108, Sinjar Formation, Kalka Smaq section. View from D, showing the EDS, Energy Dispersive spectroscopy image with elements Si and Ca representing the quartz and calcite minerals.

It is recorded in the lower part of the Sinjar Formation in the Kalka -Smaq section and the middle well-bedded unit of the type section of Sinjar Formation (Fig. 3). This facies comprises about 30–35% of bioclasts (Fig. 7A) and are mostly composed of benthonic foraminifera shells *Suadia Labrinthica*, Miliolids, Rotaliids, Textulariids, Valvulinids, in addition to bioclasts of green algae, echinoids spines, gastropods and cephalopods.

##### 4.3.2.2 Miliolidae lime wackestone submicrofacies (SMF4*)*

**Fig. 8:**
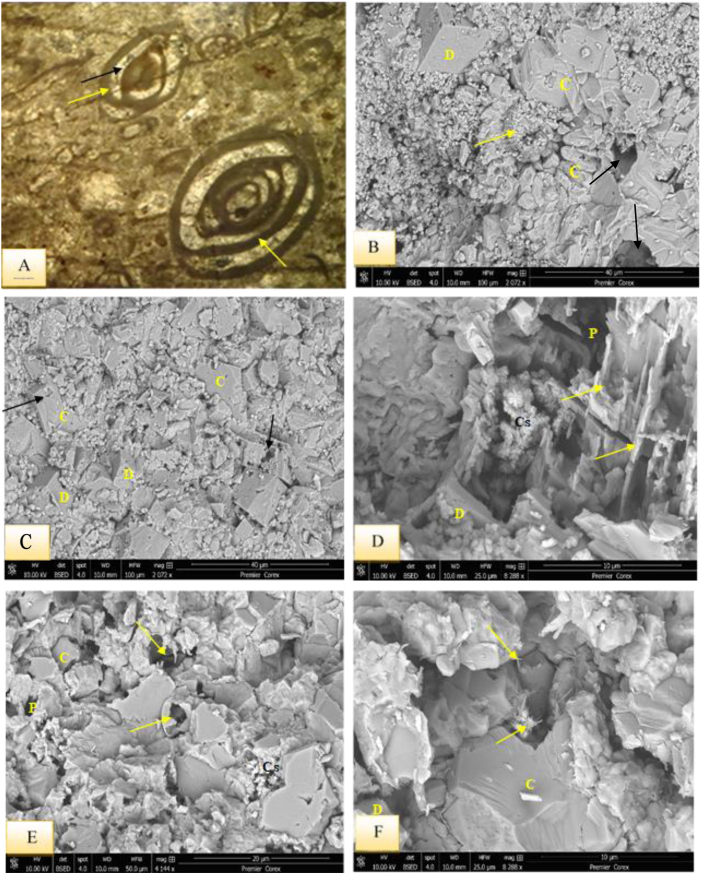
A- Miliolidae Lime Wackestone Submicrofacies and bioclasts, note cementation (black arrow) and micritization process (yellow arrows), sample 99, Sinjar Formation, Kalka Smaq section. B- SEM image from Miliolidae lime wackestone showing miliolid (yellow arrow) in micritic groundmass and dolomite (D), note the recrystallization of calcite (C) and pores (black arrows) due to dissolution, sample 100, Sinjar Formation, Kalka Smaq section. C- enlarged view of B, note common dolomite (D), hexagonal and columnar calcite that affected by dissolution (arrows). D- SEM image from Miliolidae lime wackestone from sample 110, Sinjar Formation, Kalka Smaq section showing common dissolution (arrows), vuggy porosity formation (P) with star-shaped calcite (Cs) and dolomite (D) affected by dissolution. E- moldic porosity (arrows) in SEM image, vuggy porosity (P) and common calcite in hexagonal (C) and star-shaped (Cs) forms sample 110, Sinjar Formation, Kalka Smaq section. F- SEM image for palygorskite fibers (arrows) accompanied with dolomite (D) and calcite (C), sample 110, Sinjar Formation, Kalka - Smaq section.

This submicrofacies is mainly present in the upper part of the Kalka Smaq section and the middle well-bedded unit of the Sinjar type section (Fig. 3). It is dominated by presence of benthonic foraminifera (miliolids), which make up about 35–40% of the microfacies constituents (Fig. 8A). Among the identified genera are *Triloculina, Spirolina*, *Idalina sinjarica*, *Spiroloculina*, *Quinqueloculina*, *Pyrgo*, as well as Rotaliids, Textulariids, Valyulinids, and few percentages of gastropods, cephalopods, green algae (Codacya), and bryozoans bioclasts.

##### 4.3.2.3 Dasycladacean lime wackestone submicrofacies (SMF5)

**Fig. 9:**
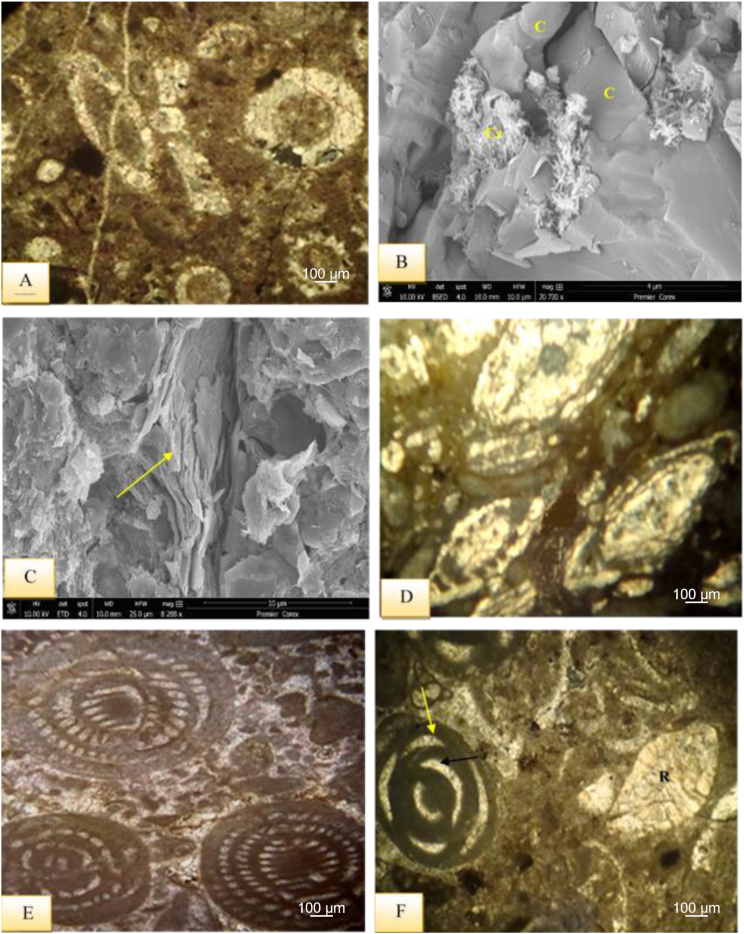
A- Dasycladacean Lime Wackestone Submicrofacies showing algae and their bioclasts in micritic groundmass, sample 85, Sinjar Formation, Kalka Smaq section. B- SEM image of Dasycladacean lime wackestone with presence of star-shaped calcite (Cs) and granular cement of calcite (C), sample 113, Sinjar Formation, Kalka Smaq section. C- SEM image for the same sample, note irregular flakes of illite/mica (arrow). D- Nummulitic Lime Wackestone Submicrofacies with bioclasts, sample 105, Sinjar Formation, type locality section. E- Alveolinidal Lime Wackestone Submicrofacies, sample 45, Sinjar Formation, type locality section. F- Miliolidae- Rotalide lime wackstone Submicrofacies showing miliolids (M), Rotaliids (R) with cementation (black arrow) and micritization (yellow arrow), sample 74, Sinjar Formation, type locality section.

This facies is present in the middle and upper parts of the Sinjar Formation of the Kalka Smaq section and in the middle well-bedded unit of the Sinjar type section (Fig. 3). Dasycladacea green algae with few amounts of benthic foraminifera and their bioclasts form the main components of this facies (Fig. 7, Fig. 9A–C).

##### 4.3.2.4 Nummulitic lime wackestone submicrofacies (SMF6)

This facies is found in the lower part of the transitional zone between Kolosh and Sinjar formations of the Sinjar Formation at Kalka Smaq section and in the upper massive unit of the Sinjar type section (Fig. 3). Up to 35% of this facies’ constituents is composed of nummulites with few ratios of *Alveolina* - and rotalids bioclasts in micritic groundmass (Fig. 9D).

##### 4.3.2.5 Alveolinidal lime wackestone submicrofacies (SMF7)

This facies is present in the lower part of the Sinjar Formation of the Kalka Smaq section and in the middle well-bedded unit of the Sinjar type section (Fig. 3). Large-size alveolinida is common in this facies, forming about 40% of the constituents in addition to green algae and their bioclasts embedded in micritic and partly microspar groundmass (Fig. 9E).

##### 4.3.2.6 Miliolidae-Rotalide lime wackstone submicrofacies (SMF8)

This microfacies is rarely found in Sinjar Formation of the Kalka Smaq section (Fig. 3B). It contains abundant miliolids and rotalids (Fig. 9F) in addition to bioclasts of cephalopods as well as echinoids and common pyrite.

##### 4.3.2.7 Interpretation

The presence of wackestone indicates a depositional environment with low to medium energy currents [38]. The occurrence of bioclasts without any preferred orientation suggests that there was no current reworking and the deposition occurred at shallow depths below the weather wave base, indicating a suitable environment for a restricted platform interior, such as a back reef setting [30,31]. The frequent presence of miliolids, dasycladacea green algae, gastropods, cephalopods, and echinoid spines in the wackestones with a micritic groundmass may indicate the deposition within FZ-7 and SMF-18, according to Refs. [30,31], respectively, further suggesting deposition in a restricted platform (back reef) setting. The minerals dominating this succession are calcite and Mg calcite, with traces of quartz (Fig. 7C–E). In restricted conditions, such as lagoons, dolomite, along with palygorskite and various forms of calcite, such as hexagonal, star-shaped, and columnar forms, could be observed (Fig. 6, Fig. 7, Fig. 8B–F), [36,39].

The occurrence of wackestone containing bioclasts of nummulite, *Alveolina* - and rotalids within a micritic groundmass may indicate deposition in platform margin reefs (fore reef setting) corresponding to FZ-5 and SMF-12 as suggested by Refs. [30,31], respectively.

#### 4.3.3 Lime packstone microfacies

This facies is characterized by the presence of 50–90% of skeletal components mainly of benthic foraminifera (Rotaliids, Textulariids, Miliolids, Valvulinids) in addition to green calcareous algae and bioclasts, commonly well-packed and grain-supported in micritic groundmass. According to its bio-content, it is further divided into six submicrofacies.

##### 4.3.3.1 Bioclastic lime packstone submicrofacies (SMF9)

**Fig. 10:**
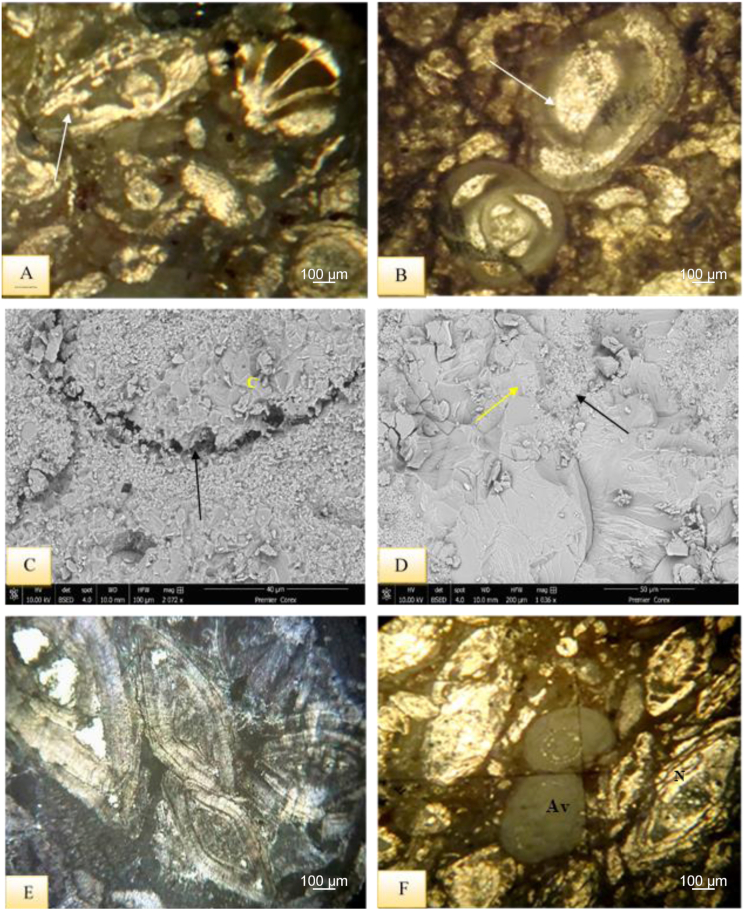
A- Bioclastic Lime Packstone Submicrofacies**,** note elongation of components (arrow) due to compaction, sample 42, Sinjar Formation, Kalka Smaq section. B- Miliolidae Lime Packstone Submicrofacies. Note also elongated miliolid (arrow) due to compaction, sample 80, Sinjar Formation, Kalka Smaq section. C- SEM image of the previous sample illustrating dissolution along miliolid shell (arrow) and recrystallization of calcite (C). D-Enlarged view of C revealed recrystallization from microspar to spar (arrows). E- Nummulitic Lime Packstone Submicrofacies, sample 115, Sinjar Formation, type section. F- Nummulitic*- Alveolina* - Lime Packstone Submicrofacies, note presence of *Nummulites* - (N) and *Alveolina* - (Av), sample 104, Sinjar Formation, type section.

This facies is reported in the lower part of Sinjar Formation in Kalka Smaq section and in the upper part of the formation at its type section (Fig. 3). It is composed of bioclasts of benthonic foraminifera (Rotaliids, *Nummulites* - *Alveolina* a- and *Disocylina* -) and red algae clasts forming about 60% of the total components embedded in micritic groundmass (Fig. 10A).

##### 4.3.3.2 Miliolidae lime packstone submicrofacies (SMF10)

This microfacies exists in middle to upper parts of Sinjar Formation in Kalka Smaq section and in the middle part of the formation at its type section (Fig. 3). It is composed of miliolids (60–70%) as a main skeletal grain (Fig. 10B–D). The identified genera are *Triloculina, Quinqueloculina, Idalina sinjarica, Spiroloculina*, in addition to *Rotalia, Valvulina, Textularia,* cephalopods, echinoderm bryozoans and green algae.

##### 4.3.3.3 Nummulitic lime packstone submicrofacies (SMF11)

This facies is present in the lower part of Sinjar Formation in Kalka Smaq section and in the upper part of the formation at its type section (Fig. 3). Nummulite skeletal grains form about 55–65% of the total facies (Fig. 10E). Components in addition to bioclasts of echinoids, Rotaliids and *Disocyyclina* - in micritic and microsparite groundmass.

##### 4.3.3.4 Nummulitic-Alviolina lime packstone submicrofacies (SMF12)

This microfacies concentrated in the lower part of Sinjar Formation in Kalka Smaq section only (Fig. 3B). It is dominated by nummulite and *Alveolina* - shells and their bioclasts in micritic groundmass (Fig. 10F).

##### 4.3.3.5 Dasycladacean lime packstone submicrofacies (SMF13)

**Fig. 11:**
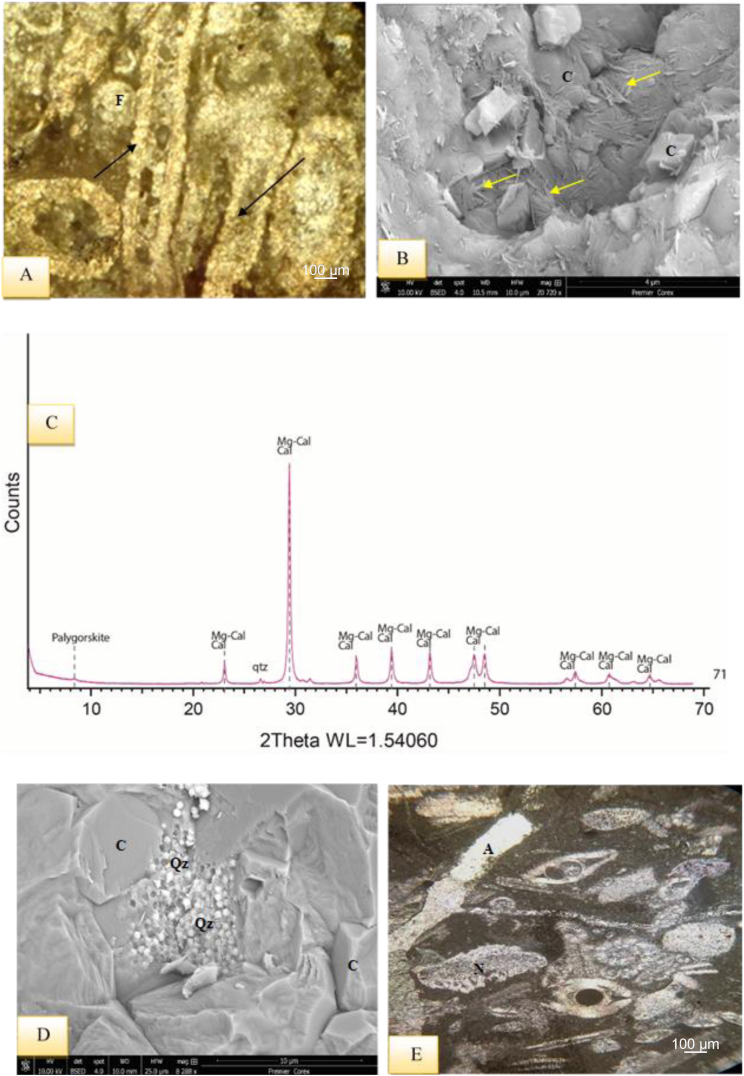
A- Dasycladacean Lime Packstone Submicrofacies including Dasycladacea algae (arrows)and foraminifera (F) with cementation inside fossil shell, sample 71, Sinjar Formation, Kalka Smaq section. B- SEM image of the same sample illustrating calcite cementation (C) and common palygorskite fibers (arrows. C- XRD diffractgram of the same sample shows dominant calcite and Mg-calcite in addition to few palygorskite and quartz. D- SEM of the previous sample showing calcite cementation (C) and quartz authigenesis (Qz). E- Discocyclina-Nummulitic Packstone Submicrofacies with *Discocyclina* algae (A) and *Nummulites* -(N) in micritic groundmass, note elongation of fossils due to compaction, sample 113, Sinjar Formation, type locality section.

It is present in the middle part of Sinjar Formation in Kalka Smaq section (Fig. 3B). It is composed of green algae (Dasycladacea) forming 60–90% of the facies (Fig. 11A), in addition to milliods and other benthonic foraminifera such as Rotaliids, Textulariids and Valvulinids in micritic groundmass. Calcite cementation and quartz authigenesis with presence of palygorskite are common (Fig. 11B–D).

##### 4.3.3.6 Discocyclina-Nummulitic packstone submicrofacies (SMF14)

This microfacies is found in the upper part of Sinjar Formation in its type section (Fig. 3A). And it is composed of nummulites and Dasycladacean (Fig. 11E), forming up to 30% of the total components in addition to alveolinids, rotaliids, red algae and milliolids embedded in micritic groundmass with common bioturbation.

##### 4.3.3.7 Interpretation

The presence of bioclastic packstone and highly micritized bioclasts and fossil shells along with association of packstone-wackstone rich in milolids and green algae represent a mostly low energy depositional environment with little impact from storm events, such as the back reef flank [38,40]. This facies falls under the FZ7 of [31] and SMF18 of [30] and suggests deposition in restricted platform environment.

However, the presence of nummulites and *Alveolina* -in packstone facies may refer to deposition in slope (fore reef setting) with high energy setting. This facies correlates with SMF 4 of [30,31] standard microfacies.

#### 4.3.4 Grainstone microfacies

This facies is a grain-supported facies and is found in the upper part of the Sinjar Formation in both Kalka Smaq and type sections (Fig. 3). The main components include; peloids, large benthonic foraminifera and bioclasts in sparite groundmass. It is divided into three submicrofacies.

##### 4.3.4.1 Peloidal grainstone submicrofacies (SMF15)

**Fig. 12:**
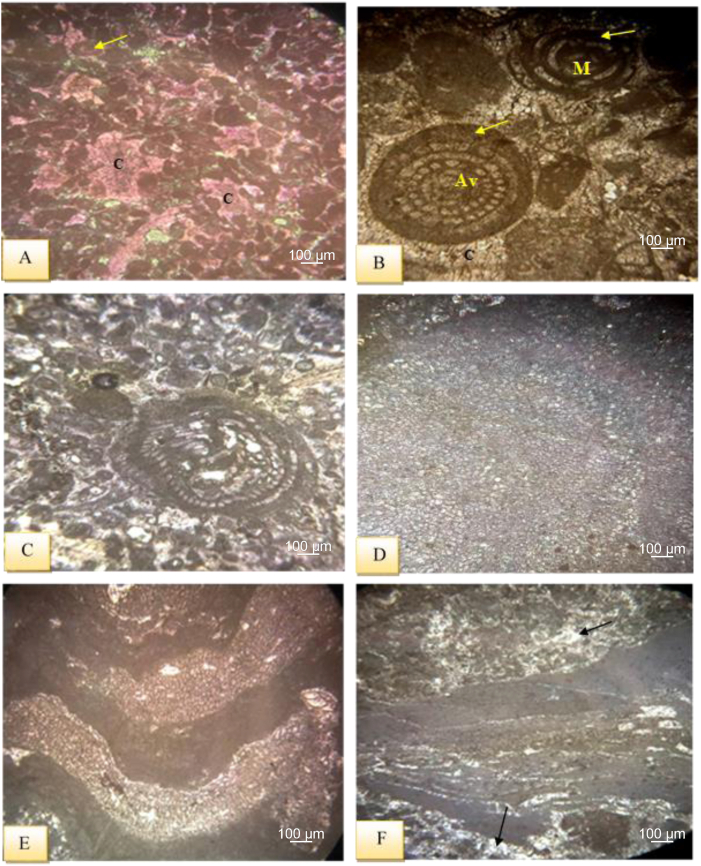
**A-** Peloidal Grainstone Submicrofacies, with common pellets and milliolids (arrow) and calcite cementation (C), sample 58, Sinjar Formation, type section. B- Alveolinal –Milliolidal Grainstone Submicrofacies, note presence of Milliolid (M), *Alveolina* (Av), micritization (arrows), calcite recrystallization (C), sample 43, Sinjar Formation, type section. C- Alveolinal-Peloidal Grainstone Submicrofacies, sample 82, Sinjar Formation, type section. D- Framstone Submicrofacies that composed mainly of coral reef, sample 91, Sinjar Formation, type section. E- Bindstone Submicrofacies, sample 97, Sinjar Formation, type section. F-Bafflstone Submicrofacies*,* note recrystallization processes (arrows), sample 99, Sinjar Formation, type section.

This facies existed in the middle well-bedded unit of Sinjar Formation type section and the upper part of the formation in Kalka Smaq section (Fig. 3). It is composed mainly of pellets in about 70% of the total facies, in addition to milliolids, *Alvolina* - and echinoids (Fig. 12A).

##### 4.3.4.2 Alveolinal–Milliolidal grainstone submicrofacies (SMF16)

This facies is reported in the middle well-bedded unit of Sinjar Formation in the type section (Fig. 3A). Large *Alvoelina* - and milliolids form about 45% of the total components in addition to few examples of rotaliids and echinoids (Fig. 12B).

##### 4.3.4.3 Alveolinal-Peloidal grainstone submicrofacies (SMF17)

This facies can be found in the upper massive unit of Sinjar Formation in the type section (Fig. 3A). Benthic foraminifera (*Alveolina* -) and pellets form the main components in this facies (Fig. 12C). Large spherical pellets as a result of micritization and small pellets are common in addition to milliolds and echinoids. Well bedding and common bioturbation are the dominant sedimentary structures in the rocks including this microfacies.

##### 4.3.4.4 Interpretation

Common peloids and milliolids found in grainstone facies may indicate deposition in shallow back-reef environments with open circulation. The presence of large-sized benthic foraminifera may suggest low salinity levels as compared to high saline restricted lagoons that is characterized by small-sized benthic foraminifera. The shallow environment is also determined by common sparitic than micritic groundmass.

This facies represents the FZ-7 of [31] and SMF12 of [30] standard microfacies which suggests toe of slope to deep shelf environments.

#### 4.3.5 Boundstone microfacies

This microfacies is constituted of in situ organisms such as, coral, red and green algae, bryozoans, with few echionoids, benthonic foraminifera and bivalves. According to Ref. [29], it could be divided into four submicrofacies.

##### 4.3.5.1 Framstone submicrofacies (SMF18)

This microfacies present in the lower part of the upper massive unit in the Sinjar Formation in the type section (Fig. 3A). It is composed of red algae and coralline algae that form 85–95% of the total components in addition to gastropods, bivalves, and bryozoans (Fig. 12D).

The common sedimentary structures are massive bedding rich in gastropod shells.

##### 4.3.5.2 Bindstone submicrofacies (SMF19)

This microfacies is reported in the uppermost portion of the Sinjar type section (Fig. 3A) and is represented by laminated algae which bound sediments as mats along with nummulites and *Disocyclina* - and red algae (Fig. 12E). Lamination and algal domal-shaped structures are the common observed sedimentary structures in the studied succession.

##### 4.3.5.3 Bafflstone submicrofacies (SMF20)

This facies is recorded in the uppermost parts of the Sinjar type section (Fig. 3A) in which the skeletal grains like coral red algae, bryozoans and echinoderms are present as columns that trap sediments and bound with other sediments during sedimentation. It forms in situ sediments that are affected by recrystallization (Fig. 12F).

##### 4.3.5.4 Floatstone submicrofacies (SMF21)

This facies is recorded in the upper part of the type section of Sinjar Formation (Fig. 3A). It mostly consists of red algae, coralline algae, and a few fragments of bryozoan in micritic and sparry groundmass affected mostly by recrystallization. Large skeletal grains form about 70% of the facies components.

##### 4.3.5.5 Interpretation

The presence of boundstone microfacies rich in coral, red and green algae, in addition to benthic foraminifera and echinoids suggest deposition in reef-building environment corresponding to facies zone FZ-5 of the standard microfacies SMF-7 after [30,31] respectively. This microfacies is represented in the current study by three submicrofacies, framestone, bindstone, and bafflestone rich in red algae, green algae and coralline algae in addition to bryozoans and echinoderms and few benthic foraminifera. The presence of framestone containing corals suggests moderate to high energy conditions, which is corroborated by the existence of gastropods and bivalves. This may indicate reef growth on platform margins, as noted by Ref. [38]. On the other hand, the occurrence of floatstone microfacies, which is rich in large corals and red algae, may suggest deposition in a slope environment.

### 4.4 Geochemistry

Paleoenvironmental studies have extensively utilized geochemical data of major and trace elements to reconstruct paleoclimates, paleosalinities, paleoredox, and paleoproductivity conditions of ancient sedimentary rocks, including sandstones, shales, and shallow-marine carbonates (e.g., Refs. [[41], [42], [43], [44], [45], [46], [47], [48]]).

Major and trace elements geochemistry of the studied succession from Kalka Smaq section have been used as geochemical proxies for paleoenvironmental interpretation of late Paleocene-early Eocene Sinjar Formation (Table 2, Table 3). Samples were taken from the transition zone between Kolosh and Sinjar formations (SK- five samples, and from Sinjar Formation, S-18 sample). Samples from Sinjar Formation represent the Paleocene –Eocene (P-E) boundary. Samples 58–95 from the Paleocene (Thanetian) while samples 100–120 from the Eocene (Ypersian), [32].

**Table 2:** Major elements (wt.%) and selected trace elements (ppm) data for limestone and marly limestone from the transition zone between Sinjar and Kolosh formations (S-K) and Sinjar Formation (S) samples. Kalka Smaq section. (See Fig. 3B for samples location). Note that the accuracy using standards Std. Hr 01 (carbonate) and Std. Hr 35 (shale).and the lower detection limit (LDL) for major elements in (%) and trace elements in (ppm) also included.

Sample	SiO_2_ %	TiO_2_ %	Al_2_O_3_ %	Fe_2_O_3_ %	MnO %	MgO %	CaO %	Na_2_O %	K_2_O %	P_2_O_5_ %	SO_3_ %	V ppm	Cr ppm	Co ppm	Ni ppm	Cu ppm	Zn ppm	Rb ppm	Sr ppm	Mo ppm	Ba ppm	Th ppm	U	Accuracy and Lower Detection Limit (LDL)			
S120	2.55	0.03	0.20	2.02	0.04	15.43	34.63	0.61	0.14	0.06	0.17	220	18	12	82	25	10	0	122	1	35	2	2	**Elements**	**Std.** **Hr01**	**Std.** **Hr35**	**LDL**
S119	8.63	0.07	0.36	2.83	0.05	17.90	26.82	0.48	0.13	0.07	0.14	294	16	17	110	18	12	0	56	0	52	2	2	**Si%**	14.07	24.2	0.1
S117	5.32	0.08	0.75	3.60	0.04	18.76	26.34	0.71	0.23	0.07	0.17	306	23	16	74	13	20	1	65	1	86	2	2	**Ti%**	0.1	0.3	0.001
S113	6.93	0.02	0.88	1.42	0.03	1.02	48.31	0.15	0.15	0.07	0.16	21	32	24	224	40	21	2	43	3	0	1	2	**Al%**	2.7	7.8	0.2
S112	0.62	0.00	0.13	0.35	0.03	0.97	53.08	0.10	0.13	0.05	0.14	0	19	1	45	22	9	2	323	6	13	1	2	**Fe%**	1.58	5.2	0.12
S110	0.63	0.01	0.12	0.42	0.03	1.93	50.04	0.14	0.14	0.05	0.15	7	19	2	46	16	8	0	345	14	21	1	4	**Mg%**	0.56	0.9	0.17
S108	2.41	0.01	0.42	1.08	0.03	1.44	50.42	0.17	0.14	0.04	0.14	17	25	9	76	19	13	0	141	6	0	1	3	**Ca%**	21.35	1.7	0.5
S100	1.72	0.01	0.22	0.53	0.03	1.15	51.66	0.12	0.15	0.05	0.14	0	21	3	44	14	9	0	216	1	24	1	5	**Na%**	0.34	0.6	0.02
S95	1.80	0.02	0.32	0.78	0.03	2.48	47.86	0.15	0.14	0.06	0.17	30	22	5	70	14	12	2	239	10	41	1	3	**K%**	0.61	3.1	0.1
S90	0.56	0.00	0.13	0.33	0.03	1.07	52.81	0.11	0.13	0.04	0.14	0	18	1	42	16	8	1	241	7	3	1	3	**P%**	0.25	0.002	0.001
S85	2.38	0.02	0.27	1.21	0.03	6.31	38.68	0.25	0.15	0.05	0.19	112	25	7	65	23	13	0	267	5	11	1	4	**S%**	1.33	2.9	0.05
S84	0.71	0.00	0.16	0.46	0.03	1.11	52.66	0.10	0.13	0.05	0.15	0	18	2	53	13	9	0	225	4	10	1	2	**Mn ppm**	94.01	252.8	100
S80	0.81	0.01	0.19	0.47	0.03	1.34	51.86	0.11	0.15	0.05	0.19	0	19	2	49	19	12	0	216	5	33	1	2	**V ppm**	280.0	459	5
S77	4.22	0.01	0.45	1.18	0.02	1.80	48.05	0.22	0.21	0.06	0.59	34	25	10	82	28	15	6	312	13	9	1	6	**Cr ppm**	76.80	110	4
S71	2.64	0.01	0.43	0.63	0.03	1.73	49.27	0.15	0.19	0.06	0.28	25	23	5	79	17	10	1	358	8	38	1	4	**Co ppm**	9.0	24	1
S66	1.38	0.00	0.26	0.56	0.03	1.68	50.54	0.11	0.14	0.05	0.26	6	19	3	49	17	11	0	281	8	13	1	3	**Ni ppm**	54.1	99	4
S63	3.28	0.04	0.52	1.57	0.03	11.89	39.07	0.34	0.18	0.05	0.21	189	26	9	80	16	13	0	324	2	56	1	3	**Cu ppm**	66.1	133	5
S58	7.75	0.05	0.93	1.93	0.02	6.86	34.48	0.35	0.28	0.07	1.26	115	26	15	91	24	17	6	203	4	96	2	2	**Zn ppm**	136.3	66	4
S-K52	16.43	0.08	1.00	3.27	0.05	15.42	24.94	0.80	0.12	0.09	1.02	254	15	23	152	15	16	0	212	1	82	2	1	**Rb ppm**	29.6	185	1
S-K46	4.94	0.06	0.58	1.53	0.03	15.21	34.28	0.76	0.19	0.08	0.21	248	24	6	40	7	13	3	124	1	131	2	2	**Sr ppm**	605.9	136	5
S-K35	8.45	0.08	0.97	3.05	0.05	17.74	26.03	0.70	0.39	0.08	1.71	309	17	17	113	17	22	8	257	9	160	2	3	**Mo ppm**	55.3	180	0.5
S-K34	3.11	0.02	0.43	0.92	0.03	3.59	44.17	0.13	0.14	0.05	0.17	44	22	6	47	17	13	1	345	1	1	1	2	**Ba ppm**	840.5	2667	5
S-K24	7.30	0.06	0.95	1.72	0.03	5.36	37.11	0.26	0.30	0.07	0.17	119	32	15	79	21	20	2	307	2	41	1	2	**Th ppm** **U ppm**	2.3 9.1	11 29	0.5 0.5

**Table 3:** Paleoenvironmental sensitive ratios for limestone and marly limestone from the transition zone between Sinjar and Kolosh formations (S-K) and Sinjar Formation (S) samples. Kalka Smaq section. (See Fig. 3B for samples location).

Sample	Paleoredox Proxies			Paleoclimate Proxies			Paleosalinity Proxy	Paleoproductivity Proxies	
	V/(V + Ni)	V/Ni	U/Th	C-value	Sr/Cu	Rb/Sr	Sr/Ba	P/Ti	P/Al
S120	0.73	2.66	0.99	1.74	4.81	0.00	0.20	1.99	0.39
S119	0.73	2.67	1.06	3.16	3.07	0.00	0.12	0.67	0.14
S117	0.80	4.11	0.81	2.31	4.92	0,02	0.06	0.76	0.12
S113	0.09	0.10	1.34	3.86	1.08	0.04	0.00	0.77	0.03
S112	0.00	0.00	2.01	0.18	14.65	0.01	1.71	33.57	0.44
S110	0.14	0.16	3.98	0.19	21.75	0.00	1.43	10.95	0.62
S108	0.18	0.22	2.80	0.72	7.59	0.00	0.00	5.22	0.14
S100	0.00	0.00	4.88	0.24	15.94	0.00	0.69	3.76	0.18
S95	0.30	0.43	2.17	0.41	16.94	0.01	0.32	3.88	0.20
S90	0.00	0.00	2.28	0.22	15.27	0.00	6.90	0.00	0.48
S85	0.63	1.72	2.85	0.68	11.73	0.00	0.90	2.93	0.15
S84	0.00	–	3.03	0.27	17.20	0.00	1.45	0.00	0.36
S80	0.00	–	1.32	0.25	11.37	0.00	0.74	5.99	0.23
S77	0.29	0.41	3.94	0.42	11.17	0.02	0.89	3.14	0.06
S71	0.24	0.31	3.51	0.30	20.57	0.03	0.46	3.12	0.11
S66	0.10	0.12	2.82	0.23	16.77	0.00	21.6	29.17	0.19
S63	0.70	2.35	1.99	0.73	20.70	0.03	0.17	0.82	0.08
S58	0.56	1.26	0.99	0.75	8.57	0.03	0.06	0.66	0.04
S-K52	0.63	1.67	0.62	1.38	14.15	0.00	0.08	0.48	0.05
S-K46	0.86	6.21	0.98	1.10	17.02	0.00	0.05	0.54	0.06
S-K35	0.73	2.72	1.27	1.02	14.93	0.00	0.04	0.50	0.05
S-K34	0.49	0.95	1.87	0.31	20.55	0.00	6.40	1.59	0.10
S-K24	0.60	1.51	1.60	0.65	14.39	0.00	0.13	0.56	0.04

**Fig. 13:**
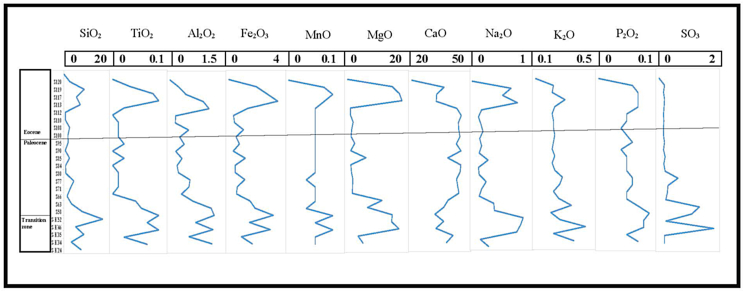
Major elements oxides (%) along the sedimentary log of the Sinjar Formation at Kalka Smaq section (See Fig. 3B for samples location).

Distribution of major oxides shows a distinguished variation between the transition zone between Kolosh and Sinjar formations and the overlying Paleocene-Eocene succession of the Sinjar Formation. All major oxides have high values in the transition zone as compared with the Paleocene Sinjar rocks. Higher values of the major oxides SiO_2_, TiO_2_, Al_2_O_3,_ Fe_2_O_3_ and MgO in the transition zone (Table 2 and Fig. 13) may reflect the contribution of detrital influx from the Kolosh Formation and the common presence of ferromagnesian minerals in the Kolosh Formation [49].

The average of SiO_2_, TiO_2_, Al_2_O_3_ and CaO in the Paleocene and Eocene samples are 2.55, 0.01, 0.37, 46.5 and 3.6, 0.03, 0.39, 34.1 respectively (Table 2). It is worth mentioning that The sudden rise in major oxide concentrations (SiO_2_, TiO_2_, and Al_2_O_3_) and the decrease in CaO amount throughout the P–E transition could be related to the dissolution of CaCO_3_ brought on by higher ocean acidity [50]. This is also supported by a slight increase in quartz and clay minerals with decrease in Mg-calcite as a common carbonate mineral in the studied rocks (Table 1).

#### 4.4.1 Paleoredox proxies

Trace elements such as V, Mo, U, and Ni, due to their insolubility in reducing environments, are sensitive indicators of redox conditions. These elements are often enriched in sediments deposited in an anoxic environment, while they are deficient in sediments deposited in oxic conditions because of their high solubility [[51], [52], [53], [[54]]. Vanadium (V), which is frequently enriched in reducing sediments, is employed in the current study as a redox-sensitive element [41,52,55]. The V/(V + Ni) ratio is thought to be a crucial proxy for paleoredox reconstruction since it shows lower oxygen regimes than other paleoredox indicators, according to Ref. [56]. In general, an environment is considered reducing if the ratio is greater than 0.5, and oxidizing if the ratio is less than 0.5 [57].

**Fig. 14:**
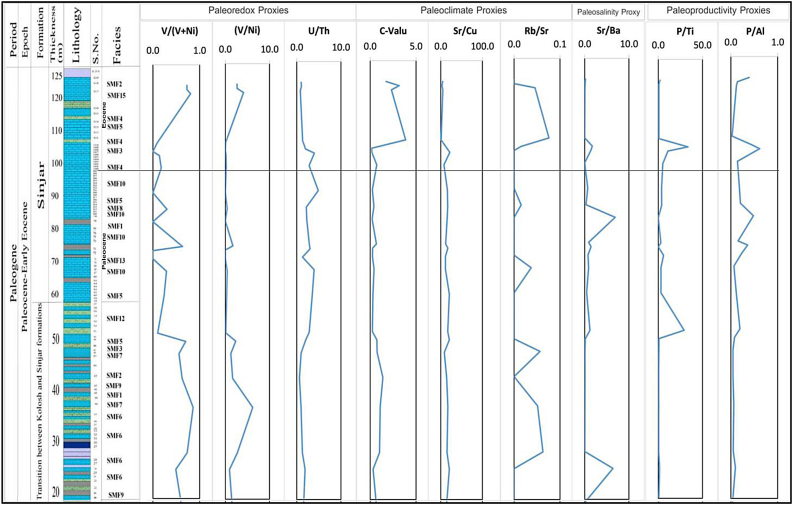
Distribution of the values of paleoredox, paleoclimate and paleosalinity proxies along the sedimentary log of the Sinjar Formation at Kalka Smaq section (See Fig. 3B for samples location).

The average of V/(V + Ni) value at the transition zone between the Kolosh and Sinjar Formation in Kalka Smaq section is 0.66, whereas, it is 0.31 at Sinjar Formation. In Sinjar Formation, the lowest 10 samples (58–95) representing the Paleocene have an average of 0.28 while the upper 8 samples (100–120) that represent the Eocene have an average of 0.33 (Table 3, Fig. 14).

The kind of environment for organic matter deposition and paleoenvironmental redox conditions are also ascertained using the V/Ni ratio [58]. V/Ni ratios: >3 indicate the deposition of marine organic matter in anoxic conditions; 1.9–3 indicate the deposition of mixed continental and marine organic matter in disoxic–oxic conditions; ratios value <1.9 reveal the deposition of terrestrial organic matter in oxic conditions.

Table 3 and Fig. 14 illustrate the elemental ratio, the average of V/Ni in the transition zone between the Kolosh and Sinjar Formation in Kalka Smaq section is 2.61, whereas, it is 0.91 at Sinjar Formation, with an abrupt increase in the uppermost three samples of the formation with as average of 3.14.

In a similar manner, paleoredox conditions of the depositional environment are also determined by the U/Th ratio. The >1.25 ratio suggests anoxic environments, ratio <0.75, indicate deposition in oxic environments [41,59].

The distribution of U/Th ratio observed in the examined samples indicates that the ratio is 1.26 in the transition zone, but it increases to 2.37 in the entire Sinjar Formation. Additionally, there is a slight increase from 2.23 to 2.49 in the Paleocene to Eocene samples, respectively (Table 3).

The abovementioned results may suggest presence of an Oceanic Anoxic Events (OAEs) in the studied succession similar to those recorded in many studies on P-E boundary globally [60].

#### 4.4.2 Paleoclimate proxies

Since trace elements remain mostly stable throughout weathering processes, they may be helpful in determining the depositional environments (see, for example, [45,[61], [62], [63], [64], [65], [66], [67], [68]]). The relative concentration of certain major and trace elements can also be used to determine paleoclimatic conditions [64,69].

In the current study, the C- value [Σ (Fe + Mn + Cr + Ni + V + Co)/Σ(Ca + Mg + Sr + Ba + K + Na) [70]; is considered as a climate proxy for interpreting paleoclimate where the standard C-values of 0–0.2, 0.2–0.4, 0.4–0.6, 0.6–0.8, 0.8–1.0 indicate arid, semiarid to semi humid, semi humid and humid conditions respectively [70] (. By displaying ratios on the studied section of Sinjar Formation in Kalka Smaq section (Table 3, Fig. 14). Increasing C values (generally over 1) in the uppermost samples of the studied sections which belong to Eocene (Fig. 14) reflects the dominance of humid conditions after the common semi-arid to arid conditions throughout the Paleocene and the boundary between Paleocene to Eocene (Table 3 and Fig. 14).

The Sr/Cu and Rb/Sr ratios are regarded as key paleoclimate indicators. The Sr/Cu ratios is a sensitive indicator of paleoclimate, with high Sr/Cu ratio generally representing a hot, dry climate, whereas low ratios represent a warm, humid climate, the Rb/Sr behave in reverse [[71], [72], [73], [74]]. In general, Sr/Cu of >5.0 refers to an arid-hot climate, while a ratio of 1.3–5.0 indicates warm and humid paleoclimate conditions (Table 3, Fig. 14). The values of Sr/Cu across the Paleocene to Eocene reflect the same trend of C-values, where high values indicate arid-hot conditions prevailing through all the Paleocene and the boundary with Eocene, while low values in the uppermost Eocene samples reveal the humid conditions, which are also indicated by higher values of Rb/Sr (Table 3, Fig. 14).

The SEM and XRD investigation revealed the presence of palygorskite accompanied with dolomite and mg-calcite with illite-mica and traces of smectite (Fig. 6, Fig. 7, Fig. 9, Fig. 11C). Presence of dolomite, Mg-calcite and palygorskite commonly reflect deposition in restricted settings such lagoon and lakes in relatively evaporitic, alkaline and saline conditions suitable for the high activity of silica and magnesia [36,39,75] in arid and semi-arid climates. This is also suggested by the presence of illite which is commonly preserved in hot and arid climatic conditions [36,47].

The present study is consistent with clay mineral associations of P-E age sediments from various sections of the Tethys, which indicate a warm and humid climate during the early Paleocene and the progressive development of palygorskite. These conditions were followed by the progressive development of arid climatic conditions from the late Paleocene to the early Eocene [76].

#### 4.4.3 Paleosalinity proxies

Ba and Sr are commonly found as bicarbonate, and as salinity increases, Ba precipitates as barium sulfate (BaSO_4_), leading to a higher concentration of Sr than Ba in the water body [77]. The Sr/Ba ratio is therefore often used as an effective index for assessing paleosalinity [45,78,79], where a ratio of >1 indicates a saline marine environment, 0.6–1.0 suggests a transition from continental to marine, and <0.6 is indicative of a freshwater environment [80]. A strong correlation between salinity and climate is indicated by the similar trend seen in the Sr/Cu curves in the sections under study (Table 3, Fig. 14). A high salinity or arid climate is indicated by higher Sr/Ba values, whereas a low salinity or humid climate is suggested by lower values. According to this study, the average Sr/Ba ratio in the Kolosh to Sinjar Formation transition zone is 1.34, while the average value throughout the Sinjar Formation is 2.09. The average value of the sediment from the Paleocene interval is 0.33, whereas in the samples from the Eocene interval, it rises to 0.52 (Table 3).

#### 4.4.4 Paleoproductivity

Phosphorus (P) is a crucial nutritional component that significantly regulates the paleoproductivity of marine and lacustrine sediments, according to Ref. [81]. P_2_O_5_ concentrations are therefore frequently used to calculate paleoproductivity [43]. To learn the impact of authigenic minerals and organic matter on the phosphorus dilution, P/Ti and P/Al ratios are also utilized to evaluate variations in paleoproductivity conditions [43,82].

The values of P/Ti ratio less than 0.34 indicates low productivity, while values greater than 0.79 indicate high productivity [82]. Ratios between 0.34 and 0.79 indicate a moderate level of primary productivity. Additionally, average P/Al ratios between 0.01 and 0.03 are indicative of high salinity, as proposed by Ref. [83].

The average values of P_2_O_5_ content, P/Ti, and P/Al ratios of the transition zone samples are 0.07, 0.73, 0.06 respectively. While these ratios are 0.05, 5.9, 0.22, respectively, in the whole Sinjar samples with an abrupt change in ratios for the uppermost samples representing the Eocene interval (Table 2, Table 3 and Fig. 14).

### 4.5 Depositional environments

Lithostratigraphic and facies analysis of the Sinjar Formation in both studied sections have revealed that the formation was deposited in a shallow marine environment extending from tidal flat into reef slope back with developed reef environment including back reef, reef core and fore reef environments.

The back reef is a shallow marine setting that extends from the shoal to the reef body including the lagoonal settings. By comparing the facies of the current study with the standard facies zones of [31] and standard microfacies of [30]; the Sinjar facies lie within facies zones FZ-6, FZ-7 and FZ-8 that extend from the shelf margin to restricted platform interior. The common microfacies in these facies zones are mudstone, wackstone and packstone. The lagoonal environment also could be divided according to their facies to inner, middle and outer lagoons [84]. The current study has revealed that the Sinjar Formation in the lower unit of the type section and in the lower part of the formation at Kalka Smaq section may represent deposition in inner lagoonal environment due to common existence of unfossiliferous and fossiliferous lime mudstone submicrofacies (SMF1, 2) rich in benthonic foraminifera, bivalves and milliolids and bioclastic wackstone. These facies zones are similar to those recorded by Ref. [84], from Mollarcoa Island in Spain, deposited in back reef settings. The presence of dolomite with palygorskite as indicated by XRD and SEM support the deposition in restricted saline lagoonal environment [36,39]. This is also supported by high paleosalinity proxy (Sr/Ba) especially in the lower part of the Sinjar Formation (see sample S66, Table 3 and Fig. 14) which records the highest Sr/Ba ratio, an indicative of high saline and evaporative conditions in this facies responsible for the presence of dolomite and palygorskite authigenic minerals [36,39]. However, paleoclimatic proxies also suggest arid hot paleoclimate with common illite in the lower part of the Sinjar Formation (Table 1, Table 3 and Fig. 14) where these microfacies, indicative of lagoonal environments are common. The arid to semi-arid conditions prevailing also during the middle part of the Sinjar Formation (Paleocene interval samples), while the upper most part of Sinjar Formation (Eocene interval samples), the prevailing conditions were humid as indicated from the paleocliamtic geochemical proxies.

In terms of paleoredox conditions, it is well known from geochemical study, that reducing conditions dominate the transition zone samples which represent the black shale and marl while oxidizing conditions prevailed during deposition of the Sinjar Formation except the uppermost part of the formation including the Paleocene-Eocene interval which reflects prevailing anoxic conditions with a gradual depletion of oxygen during the deposition of the Eocene interval samples (Fig. 14).

The depositional environment of the Sinjar Formation in the middle well-bedded unit of the type locality and the upper part of the Kalka Smaq section is suggested to be middle lagoon. This is supported by the dominance of milliolid wackstone, Rotalid-milliolid wackstone, Dasycladacean wackstone, milliolid packstone, and Dasycladacean packstone facies. The occurrence of these facies in thick limestone layers containing abundant large milliolid and small rotalid shells suggest that they were deposited in a sheltered back reef environment, possibly a middle lagoon [85]. Moreover, green algae belonging to the Dasycladacea group have been identified in many back reef environments located in tropical and subtropical regions [30] as well as in several areas in Europe [86]. The successions of the Sinjar Formation in the middle and upper parts of the Kalka Smaq section and the uppermost beds from the type locality section were deposited in the outer lagoon environment which is dominated by grainstone facies rich in benthonic foraminifera, red algae and bioclasts of bryozoans and echinoids similar to those identified by Ref. [15] from the Sinjar Formation in northwestern Iraq.

The rocks deposited in back reef setting are characterized by their diverse living organism due to varying water energy, salinity, depth, light transmittance and nutrient. Benthonic foraminifera such as Miliolids, Valvulinids, Peneropilids in addition to red algae, gastropods and cephalopods in the present study are similar to those recorded in several studies on the Paleocene and Eocene carbonates, such as offshore Tunisia [87] and patch reefs of Bermuda [88] and the presence of Dasycladacean green algae in the Eocene succession in various localities in Europe, such as Belgium, Slovenia, France and England [86].

Geochemical proxies of paleoproductivity suggest that primary productivity increased significantly, especially during the early Eocene, which led to an increased supply of nutrients, possibly due to enhanced upwelling, and is evidenced by the abundance of benthic foraminifera and other bio content in the Sinjar Formation, especially across the P-E boundary. The study region is part of the southern margin of Tethys, which has seen periods of upwelling events and a notable atmospheric contrast between the humid, tropical and arid, sub-tropical conditions [21].

The reef and fore reef environments were recorded in the upper part of the Sinjar Formation in the type section and are characterized by presence of boundstone (framestone, bindstone, and bafflestone) facies, in addition to nummulitic packstone facies. The last facies refers to shallow shoal in fore reef environment [89]and the presence of bioclasts of coralline algae, red algae and coral accompanied with nummulites are good indicators for breakup from the reef core and redeposit in the fore reef setting [90].

The presence of boundstone facies with their coral, coralline algae and red algae components is one of the main reef core facies [90,91]. Reefal rocks commonly form massive beds rich in red algae, green algae, benthonic foraminifera, gastropods, bivalves and echinoids and are restricted between near-shore back reef and fore reef settings in shallow depths [92]). According to Ref. [84], the grainstone facies rich in bioclasts, red algae and benthonic foraminifera commonly exist in reefs near to fore reef setting while floatstone facies present in channel within reef (reef front). Similar facies assemblages were recorded from the Eocene-Miocene carbonates in Jabal a1 Akhdar (northeast Libya) which consist mainly of bioclastic packstone, nummulitic packstone, nummulitic-*Alvolina* - and dasycladacean packstone facies in addition to nummulitic and dasycladacean wackstone facies [93].

In the current study, the lower part of the Sinjar Formation at Kalka Smaq section and the upper part of the formation at type section were deposited in shallow marine fore reef (shoal) similar to those previously mentioned for the Sinjar Formation by Refs. [15,94,95] who recorded the shoal barrier environment rich in nummulite and dasycladacea for the formation.

The slope environment of the Sinjar Formation is suggested by the presence of bioclastic packstone facies in the lower part of the formation at Kalka Smaq section and floatstone facies in the middle and upper units of the formation at its type section.

According to these data, the Sinjar Formation was deposited in a shallow marine environment extending from back reef to fore reef with their sub environments. The reef core and fore reef environments, the main environments in the Kalka Smaq section, are not well-developed as compared to those in the type section of Sinjar Formation and may relate to the location of this section at the margin of Sinjar Basin as recorded from the intercalations with the underlying Kolosh Formation in the transitions zone (Fig. 3, Fig. 5).

**Fig. 15:**
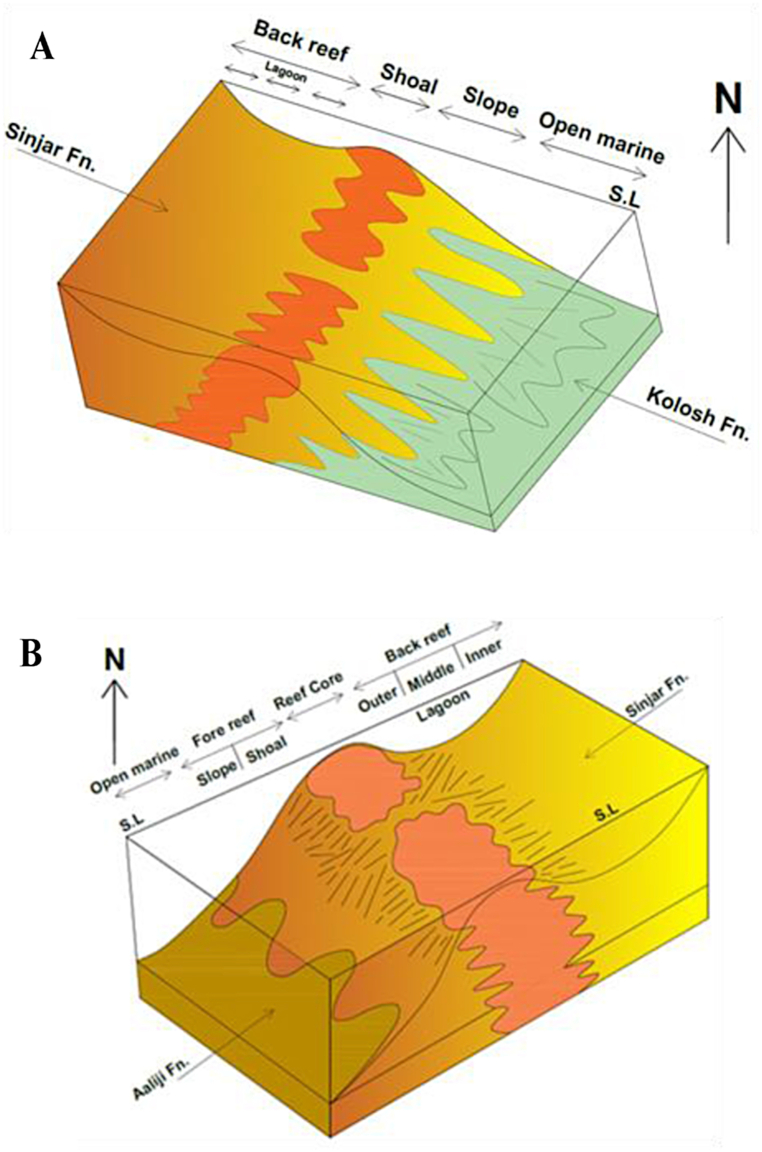
Models represent (A) Succession of Sinjar and Kolosh formations in the Kalka Smaq section including the interfingering transition zone between the two formation and the shoal of the Sinjar Formation. (B) Well developed reef succession at Sinjar type section including the Sinjar shoal separating Sinjar Formation from the Aaliji Formation.

Upon comparison of the studied environments with the sedimentary models proposed by Ref. [96], it was observed that the successions of the Sinjar Formation are consistent with the rimmed platform model, which is distinguished by the occurrence of a well-developed slope environment. This observation is supported by the paleogeography of the region during the Paleocene to early Eocene period when the Sinjar Formation was deposited on a shallow submarine ridge that separated two deep basins - the clastic Kolosh Basin to the northeast (Fig. 15A) and the deep carbonate Aaliji Basin to the southwest [15], (Fig. 15B).

Numerous clues to the depositional and paleoclimate changes from the Paleocene to the Eocene, including the PETM event that had an impact on the distribution of mineralogical and geochemical composition, have been found in the elemental and mineralogical variation throughout the Paleocene-Eocene Sinjar Formation. The dolomite deposition in the uppermost samples of the investigated sequence, where the Mg/Ca ratio was suitable for this deposition, is supported by an increasing amount of Mg content. Increases in SiO_2_, TiO_2_, Fe_2_O_3_, and Al_2_O_3_ content are also present along with this, and these are appropriate for the more weathering conditions that affect this area following warming conditions during the P-E transition [97,98].

Based on various geochemical and mineralogical proxies, the paleoclimate was determined for the succession of the Paleocene to Eocene Sinjar Formation in the Kalka Smaq section, which was found to be deposited under a dry and hot climate during most of the Paleocene and the P-E boundary and vary gradually to warm humid climate conditions in the upper most part of the succession representing the Eocene period. Increasing temperature in sediments of the Paleocene-Eocene boundary than those from the Paleocene was also in Iraq and globally [9,60]. In general, based on the ratios of V/(V + Ni), V/Ni, and U/Th, an indication for an Oceanic Anoxic Events (OAEs) were also recorded in the studied section were deposited in low to moderate oxygen environments, while the lack of oxygen was clearly determined in the PETM period through the evidences of environmental and climatic conditions recorded through the sedimentological, biological and geochemical indications from the current study which reflects the paleoclimate fluctuation in northern Iraq in response to the global Paleocene-Eocene transition [7,60,99].

## 5 Conclusions

Facies analysis of the Paleocene-Eocene succession of Sinjar Formation from northwestern and northeastern Iraq, has revealed that the succession including five main maicrofacies; lime mudstone, wackstone, packstone, grainstone, and boundstone that were deposited in a shallow marine environment, back reef (lagoonal, inner, middle and outer lagoon), reef core, and fore reef environments with a well-developed reef system in the Sinjar type section of northwestern Iraq than Kalka Smaq section from northeastern Iraq.

Major and trace elemental data and paleoenvironmental proxies implied for paleoredox, paleoclimate, paleosalinity and paleoproductivity across Paleocene-Eocene (P-E) transition boundary suggest normal variation form oxygenated conditions during the late Paleocene succession to anoxic conditions during the early Eocene. This is accompanied with an increase in nutrients and primary productivity due to the effect of upwelling currents during the early Eocene. Salinity levels also were changed from low to high across the P-E boundary, which can be indicated geochemically by Sr/Ba ratios or common presence of Mg-calcite, dolomite, and palygorskite. Consequently, paleoclimatic changes across the P-E transition show variation from arid to semiarid and then to humid conditions. These variations in paleoenvironmental proxies coincide with a globally recognized environmental and climatic parameters changes across P-E transition.

## Data availability

There is no research related data stored in publicly available repositories, and the data will be made available on request.

## CRediT authorship contribution statement

**Noor T. Al-Taee:** Resources, Formal analysis. **Ali I. Al-Juboury:** Writing – review & editing, Writing – original draft, Conceptualization. **Imad M. Ghafor:** Writing – original draft, Investigation. **Giovanni Zanoni:** Methodology. **Harry Rowe:** Methodology.

## Declaration of competing interest

The authors declare that they have no known competing financial interests or personal relationships that could have appeared to influence the work reported in this paper.
